# External Urethrotomy Versus Internal

**Published:** 1894-02

**Authors:** W. C. Dugan

**Affiliations:** Louisville, Ky.


### EXTERNAL URETHROTOMY VERSUS INTERNAL.*

BY W. C. DUGAN, M. D.,

LOUISVILLE, KY.

I am convinced that there is not another operation so seriously neglected as external urethrotomy, and, too, none over-magnified as to its difficulties and complications. While I shall discuss it in its general sense, I desire more especially to enter a strong plea for urethrotomy versus epicystotomy and retro-catheterization in those cases where it is impossible to introduce a guide.

It is maintained by some that so long as even a drop of urine can be forced out, the surgeon should, by patience and perseverance, be able to introduce a small instrument. While this may be true,

* Abstract of essay read before meeting of Louisville Surgical Society, November, 1893.

and I confess it is in most cases, it would be difficult to convince one that it was practicable when he had stood over a patient with all kinds of instruments at his command, and worked untiringly, without success for two and one-half hours, and finally forced to abandon it and to resort to external urethrotomy without a guide. I have had in all something near twenty cases, so I have had an experience in this operation that we are told is so very difficult and tedious, and for that reason feel warranted in giving the conclusions I have reached concerning the operation, its technique, its indications, its advantages, etc.

After a most careful study of the subject, and a review of my rather large number of cases, I am thoroughly convinced that epicystotomy is an operation of itself, too important to be considered a step in the performance of urethrotomy in those cases where the passage of the smallest instruments are not found practicable.

We are told that urethrotomy without a guide is a most difficult surgical procedure. Of this I was thoroughly convinced, up to a time when I had yet my first operation to perform. I shall never forget with what fear and trembling I approached my first perineum to operate, without having been able to introduce a sound, and the pleasant surprise experienced when the urethra was found, opened and stricture relieved. If one is ignorant of a practical knowledge of the anatomy of the perineum, I can conceive of nothing more difficult. But on the other hand, if one knows the anatomy as it is found on the subject, a knowledge which comes alone by many careful dissections and not book anatomy, I am just as positive that I know of no operation which can be more satisfactorily and scientifically performed.

I have experienced so little difficulty in doing the operation without a guide, that I confess in most cases I have not persisted (as most surgeons do) in trying to get the sound through the stricture after making up my mind to do an external operation, but place the patient in position and operate without a guide, rather than worry for an hour or more in getting into the bladder; the main question in my mind being, which of the two operations—internal or external—shall we do, rather, those with or without the guide.

Before describing the operation, it might be well to give my reason for asserting that external urethrotomy should be oftener performed. The normal urethra is a closed canal, the sides being in contact with each other. But such is not the condition of a pathological canal, when the urethra is more or less sacculated and more or less urine is retained after each micturition.

Now, with a stricture located in the deep urethra, and it is cut from within, there are pockets (numbering according to the number of strictures) in which is retained some urine from one urination to the next, provided this canal is not washed out after each flow of urine.

When we are to understand that all these cases so badly strictured as to render difficult the introduction of instruments have cystitis, and as a result the urine is filled with pus and teeming with pathogenic micro-organisms, we should expect chills, fever and other systemic disturbances produced by the absorption of this septic material which finds such a ready entrance through the incised stricture which is rich in lymphatics. The question is, why is it not the rule, rather than the exception. Antiseptic surgery has done much to lessen urethral fever, but we still meet with it, notwithstanding the strict precautionary measures we throw around all these cases.

In a few words I might sum up the advantages of external urethrotomy over internal, as I conceive them to be, to wit:

First.—The drainage is so perfect that no fluid can accumulate in the wound.

Second.—Cleansing and dressing the parts, are satisfactory in all particulars.

Third.—The patient experiences very much less pain during and after urinating, which I consider very important, since the passing of urine after internal cutting is so painful that the patient is often made to cry out, and the vesical tenesmus so great as oftentimes to cause quite a free bleeding after each micturition, for some days.

Fourth.—The certainty of opening the canal against the possibility of making false passage, as is not infrequently done in our efforts to introduce small instruments.

Fifth.—The absence of any risk of patient bleeding to endanger his life, as the field of operation is unobstructed, and the bleeding points can usually be secured and ligated, or if the bleeding is capillary, controlled by the application of compress accurately applied.

In describing the operation I shall in brief detail the various steps as I have learned to proceed in the cases that have come under my own observation. The preparation of the patient should be as carefully looked to as for other major operations. We should have at least two, and better, three assistants, besides the anesthetist. The patient should be placed in the lithotomy position and drawn quite to the edge of the table. The operator, seated on a stool of proper height and between the patient's thighs, makes his incision through the skin and superficial fascia about two inches long, being very careful to conform strictly to the raphe. He should make several free strokes with the knife and avoid making the wound funnel-shaped. No time should be lost in exposing the deep fascia which is recognized by its being rather thick and glistening and hardly to be mistaken for any other structure when we remember it is convex from side to side, conforming to the shape of the bulb of the urethra which it covers. If we should be in doubt as to the fascia, it may be incised so as to bring the muscle into view and its bipenniform appearance—fibers diverging—we cannot mistake it for any other structure. This muscle, and the fascia overlying it, is the chief deep guide in performing this operation, for they cover the bulbous urethra.

The knife should here be laid aside and a pair of forceps and blunt scissors taken, the fascia dissected up carefully next to the perineum and also along the side posteriorly; then with a stout blunt tenaculum hooked over behind the muscle and fascia, and it lifted up and drawn forward toward the scrotum (this being done to avoid cutting the bulb). I am confident that the importance of this part of the operation is not realized. With the bulb drawn forward out of the way we come at once down on the last layer of deep fascia. This should be freely incised from before backward to at least three-fourths inch, and a pair of blunt retractors caught under its free edge and the wound drawn open. When this is done and the wound cleansed by free irrigation, the membranous urethra is exposed surrounded by the constrictor urethra. The urethra should be held firmly with forceps or sharp tenaculum, and its lumen opened by cutting from without in. The urethra having been exposed and opened, the last step, cutting the stricture, is determined by its location.

If the stricture be located at the bulbo-membranous junction, we may find the membranous urethra abnormally large, in fact, sacculated, which renders easy the last step of the operation. But if the stricture is in the membranous urethra, it is best to cut down on it from without; whereas, if it is further back, at the prostato-membranous, it is best to open the canal in front and clear the field of operation thoroughly by free irrigation; then with the lips of the wound drawn well apart, we may expect but little trouble in passing a probe through the stricture into the bladder, this to be followed by a larger instrument and then by the ordinary dressing forceps passed closed through the stricture, then opened and withdrawn. Lastly, the careful introduction of the finger by a rotary or boring motion, we can dilate it up to any size desired. The dilatation should not be confined to the stricture, but the prostate as well, as it gives the patient much relief in voiding his urine, nor does it cause incontinence as is believed and taught by some. If after dilating well, it is found to contract immediately, like a rubber band, it should be cut in several places by the careful use of the straight blunt bistoury until all tendency to so close has been overcome.

If the stricture be located at the bulbo-membranous, the urethra should be opened as already described, this time behind the stricture, hemorrhage arrested, lips well drawn apart, and a careful search instituted for the contracted urethra. Here again we rarely have any difficulty in locating the small opening and passing from behind forward the filiform. I think it is preferable to cut the stricture located here. This may be accomplished by passing your Maisonneuve with Gouley tip down on the filiform either from behind forward or *vice versa*. Having passed the staff through, the stricture should be cut with the smallest knife, the incision preferably on the roof of the canal. Now the Otis should be introduced from the meatus and the stricture dilated up to tension and then cut; then close instrument, push the knife back to the end, turn urethrotome so as to cut on opposite side. Again dilate up to tension and again cut, and so on, changing instruments so as to cut a little here and a little there until the desired size is attained, say 35 F. I have found in two cases that even with the filiform passed through from behind it was still impossible to pass the urethrotome on it, so that I usually tie on to the filiform a very stout piece of cable silk, or preferably, a strong flax thread. Now the filiform is withdrawn, and along with it the thread is pulled through and left in the stricture. With this thread through the stricture, one end hanging out of the meatus and the other through perineal opening, we can readily enlarge the canal by grasping the two ends and drawing them rapidly to and fro in a saw-like manner, cutting the stricture by means of the thread. This has one very decided advantage over all other methods of cutting; there is no bleeding, or we could cut the urethra with the thread up to a size that will admit the introduction of the Otis, and complete the operation with that instrument.

If for any reason it is desired to cut with an Otis or Maisonneuve, the stout cord, having once been passed through, it should be securely tied on to the end of the urethrotome. Now, by pulling on the cord, the instrument is made to pass through the stricture without the remotest possibility of making false passage or otherwise damaging the canal, provided due care be observed in its manipulation. This I have found to work well in the few cases in which I have given it a trial.

Some prefer to introduce a drainage tube through the perineal wound into the bladder and leave it there for a number of days. This I do not consider necessary, in fact, am confident that it gives the patient much unnecessary pain and cannot possibly do him any good, hence, should, in my judgment, be dispensed with entirely. The wound should be washed frequently. The canal should be irrigated night and morning and at the same time we should carefully introduce through the perineal wound a small, soft catheter into the bladder and wash it out with a sat. sol. of boric acid, then a small pad of gauze applied against same and secured by the application of a "T" bandage. If the patient experiences much burning while micturating, it would be well to apply oil or vaseline just prior to the flow and it will give much relief. Patient should be kept in bed for one week. Bowels should be kept open and patient encouraged to take largely of liquids. By the end of the week, if patient has had no complications, I introduce the solid sounds. I find that most patients require chloroform for this operation, as I feel that it is important to pass them up to full size. As a rule I order ten grains quinine the night before the introduction of sounds. He is kept in bed two days, washed out as after operation, and if he has no trouble he is then allowed to go out. By this time he voids most of his urine through the natural channel and has but little pain. Patient is then directed to introduce the straight solid sound, size about 20 to 24 F., each night on going to bed, with instructions that he is to see me once each week to have the large sounds introduced. If this is painful, cocaine solution is sometimes used, but as a rule it is not called for. This is kept up for one month and the patient is discharged; instructed to introduce his sounds once or twice each week the rest of his life as a preventive measure.

